# Elevated lipoprotein(a) and genetic polymorphisms in the *LPA* gene may predict cardiovascular events

**DOI:** 10.1038/s41598-022-07596-4

**Published:** 2022-03-04

**Authors:** Jun-Xu Gu, Juan Huang, Shan-Shan Li, Li-Hua Zhou, Ming Yang, Yang Li, Ai-Min Zhang, Yue Yin, Na Zhang, Mei Jia, Ming Su

**Affiliations:** 1 Department of Clinical Laboratory, Peking University People’s Hospital, No. 11 Xizhimen South Street, Beijing, 100044 People’s Republic of China; 2 Department of Traditional Chinese Medicine, Peking University International Hospital, Beijing, People’s Republic of China; 3 Department of Clinical Laboratory, Peking University International Hospital, Beijing, People’s Republic of China

**Keywords:** Predictive markers, Coronary artery disease and stable angina

## Abstract

Elevated lipoprotein(a) [Lp(a)] is a risk factor for coronary heart disease (CHD), but there are few studies on the prediction of future cardiovascular events by Lp(a) and its *LPA* single nucleotide polymorphisms (SNPs). The aim of this study was to investigate whether elevated Lp(a) and its SNPs can predict cardiovascular events. We evaluated whether Lp(a) and *LPA* SNPs rs6415084 and rs12194138 were associated with the incidence rate and severity of CHD. All participants were followed up for 5 years. Elevated Lp(a) is an independent risk factor for the risk and severity of CHD (CHD group vs. control group: OR = 1.793, 95% CI: 1.053–2.882, *p* = 0.043; multiple-vessel disease group vs. single-vessel disease group: OR = 1.941, 95% CI: 1.113–3.242, *p* = 0.027; high GS group vs. low GS group: OR = 2.641, 95% CI: 1.102–7.436, *p* = 0.040). Both *LPA* SNPs were risk factors for CHD, and were positively associated with the severity of CHD (*LPA* SNPs rs6415084: CHD group vs. control group: OR = 1.577, 95% CI: 1.105–1.989, *p* = 0.004; multiple-vessel disease group vs. single-vessel disease group: OR = 1.613, 95% CI: 1.076–2.641, *p* = 0.030; high GS group vs. low GS group: OR = 1.580, 95% CI: 1.088–2.429, *p* = 0.024; *LPA* SNPs rs12194138: CHD group vs. control group: OR = 1.475, 95% CI: 1.040–3.002, *p* = 0.035; multiple-vessel disease group vs. single-vessel disease group: OR = 2.274, 95% CI: 1.060–5.148, *p* = 0.038; high GS group vs. low GS group: OR = 2.067, 95% CI: 1.101–4.647, *p* = 0.021). After 5 years of follow-up, elevated Lp(a) and *LPA* SNPs rs6415084 and rs12194138 can independently predict cardiovascular events. The increase of serum Lp(a) and *LPA* SNPs rs6415084 and rs12194138 are associated with increased prevalence and severity of CHD, and can independently predict cardiovascular events.

## Introduction

Cardiovascular disease has the highest incidence rate and mortality rate in the world^1–4^. Over the past decades, a large number of studies have reported its possible risk factors, such as diabetes, hypertension, dyslipidemia, and smoking, in order to early assess the risk of cardiovascular disease^5–8^. At present, large epidemiological and genetic studies have provided strong evidences that lipoprotein(a) [Lp(a)] is a causal risk factor for coronary heart disease (CHD)^9–11^.

Lp(a) is a lipoprotein synthesized from the liver. It is an LDL-like particle that consists of an apolipoprotein(a) moiety linked to one molecule of apolipoprotein B100 via a disulfide bond^12^. Like low density lipoprotein cholesterol (LDL-C), Lp(a) can accumulate in the subendothelial space, leading to progressive atherosclerosis^13^. It has also been shown to produce more signaling, enhance its atherosclerotic ability. Lp(a) can induce systemic inflammatory response, thrombosis and promote oxidation^14^.

Plasma Lp (a) level is mainly determined by the *LPA* gene variation encoding apolipoprotein (a)^15^. Individual Lp(a) levels range from < 0.1 mg/dl to > 200 mg/dl, and it was highly heritable^16^. *LPA* is the major gene controlling this quantitative and co-dominantly expressed trait in all populations^3^. In recent years, many single nucleotide polymorphisms (SNPs) have been found in *LPA*^17,18^, such as rs6415084 and rs12194138, were closely associated with Lp(a) levels.

The relationship between Lp(a) levels and SNPs and the risk and severity of CHD as well as future recurrent cardiac events have been less studied, but so far they seem to be weak. We aimed to systematically assess whether Lp(a) and two *LPA* SNPs are associated with the occurrence and severity of CHD and long-term cardiovascular events.

## Methods

### Study population

We enrolled 2766 Han Chinese subjects (1614 males, 1152 females) from Peking University People’s Hospital and Peking University International Hospital from May 2013 to September 2015. There were 1665 patients (993 males, 672 females) with CHD and 1101 subjects (621 males, 480 females) in the control group. The follow-up procedures were performed by experienced nurses or doctors every 6 months via telephone or face-to-face interviews. Major cardiovascular events (MACEs) are divided into cardiovascular mortality, non-fatal myocardial infarction (MI), non-fatal stroke, heart failure, hospitalized unstable angina and non-coronary heart disease patients diagnosed as coronary heart disease. The longest follow-up time is 5 years.

The diagnostic criteria for patients with CHD were based on the coronary angiography (CAG) performed in our institution, and defined as at least one major coronary artery occlusion or stenosis of more than 50% and the severity of CHD was evaluated by the Gensini score (GS). CHD patients were divided into three groups according to their GS: low GS (GS ≤ 25), intermediate GS (GS: 26–41) and high GS (GS ≥ 42). The control group who all received coronary computed tomographic angiography (CTA) was selected from the physical examination center during the same time period, and included individuals without CHD.

All patients diagnosed as type 2 diabetes were selected based on the criteria set by the American Diabetes Association: (1) self-reporting to the clinician that he/she has a history of type 2 diabetes, (2) under current treatment of oral hypoglycemic medicine or insulin, (3) repeated fasting plasma glucose (FPG) greater than 7.0 mmol/L, or (4) glycated hemoglobin A1c (HbA1c) ≥ 6.5%.

Exclusion criteria included: (1) percutaneous coronary intervention within the previous three months, (2) acute coronary syndrome within the previous six months, (3), history of coronary artery bypass operation, (4) chronic heart failure, cardiomyopathy, valvular heart disease, (5) pulmonary heart disease, (6) severe liver and kidney dysfunction, or (7) any known inflammatory or infectious disease, or confirmed or suspected cancer.

The present study complied with the Declaration of Helsinki and was approved by the Peking University People’s Hospital Research Ethics Committee. Informed written consents were obtained from all patients enrolled in this study.

### Measurements of lipoprotein(a) and other biomarkers

Blood samples were collected in the morning after at least 12 h of fasting. All measurements were performed within 6 h. Lp(a) in the serum samples was measured using latex enhanced immunoturbidimetry. The Lp(a) detection kit (Roche Inc., Germany) was used to determine the precipitation at 800/660 nm using latex particles coated with anti Lp(a) antibody to allow agglutination with human lipoprotein. FPG, homocysteine (HCY), hypersensitive C-reactive protein (hs-CRP), serum lipid profiles, including triglycerides (TG), total cholesterol (TC), LDL-C, and high density lipoprotein cholesterol (HDL-C), were analyzed with a Beckman AU5832 analyzer (Beckman Coulter Inc., USA). Apolipoproteins A-1 (apoA1) and B (apoB) were measured by immunoturbidimetry (Daiichi Pure Chemicals Co., Ltd., Tokyo). Direct quantitative analysis of small dense low-density lipoprotein cholesterol (sdLDL-C) assay was done using sdLDL-C reagent kits (Denka Seiken Co., Ltd. Japan). The HbA1c was determined with high-performance liquid chromatography (Trinity Biotech Inc., USA).

### DNA extraction and genotyping

According to the manufacturer's instructions, genomic DNA was extracted from 3.5 ml EDTA anticoagulant blood collection vessel (Becton, Dickinson and company, USA) using puregene DNA separation kit (TianGen Biotech, Beijing, China). The incidence of *LPA* variant rs6415084 or rs12194138 was determined by gene sequencing (TsingKe Biological Technology, Beijing, China). All Sanger sequencing data shall be visually inspected by three experienced operators in our laboratory.

### Statistical analyses

All subjects were matched using propensity score matching, including age, sex, body mass index (BMI), diabetes, hypertension, smoking, consumers of alcohol. All data were tested by one sample Kolmogorov–Smirnov test to determine whether the distribution of quantitative variables was normal. The normally distributed data were reported as means ± SD, and the differences between various groups were compared by the Student’s t test. The abnormally distributed continuous variables were reported as medians (interquartile range), and the differences between various groups were compared by the Mann–Whitney U test. The χ2 test was used to examine the Hardy–Weinberg equilibrium for each variant and to compare the distribution of allele and genotype frequencies between CHD patients and control subjects. The association of Lp(a) and SNPs with the presence and severity of CHD was analyzed using multivariate logistic regression adjusted for age, sex, BMI, diabetes, hypertension, smoking, consumers of alcohol, FPG, HbA1c, ApoB, ApoA1, TC, TG, HDL-C, hs-CRP, and HCY. The Kaplan–Meier method was used to estimate the event-free survival rates among groups. The significant level of all statistical tests was *p* < 0.05. SPSS 22.0 for Windows (SPSS Inc., USA) and GraphPad Prism 7 (GraphPad Software Inc., USA) were employed for the statistical analyses.

### Ethics approval and consent to participate

The present study complied with the Declaration of Helsinki and was approved by the Hospital Research Ethics Committee, and written informed consent was obtained from all patients.

## Results

### Baseline characteristics of the study group

**Figure 1:**
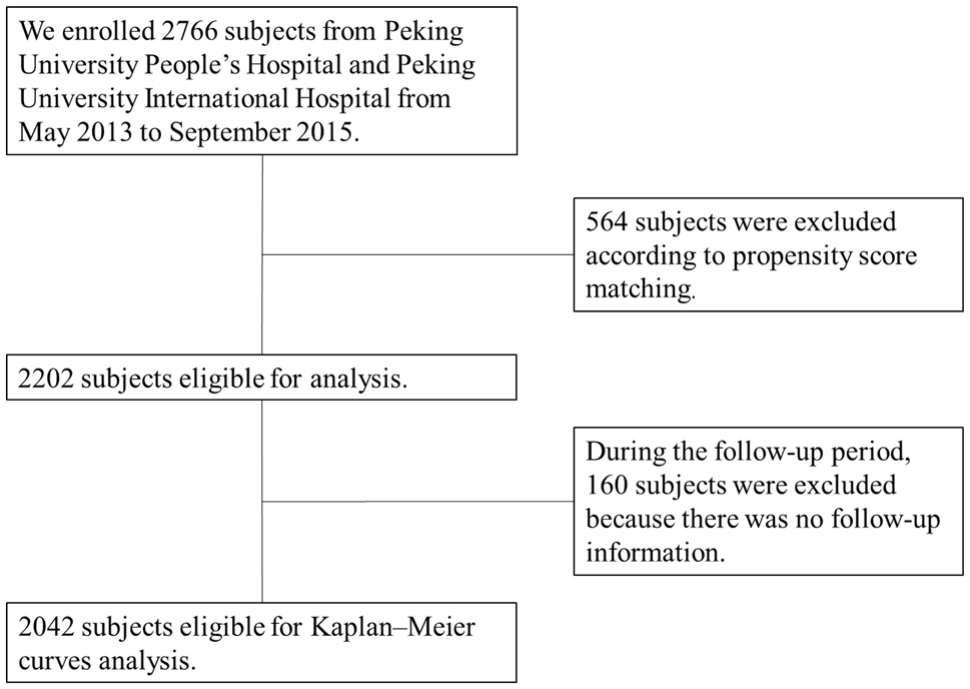
The flow chart of the patients’ selection process.

The recruitment scheme of all subjects is shown in Fig. 1.

Baseline clinical data of all subjects (Table 1). There was no significant difference in age, sex, BMI, diabetes, hypertension, smoking, consumers of alcohol, FPG, HbA1c, TC and TG between CHD group and control group. The levels of ApoB, LDL-C, sdLDL-C, hs-CRP, Hcy and Lp(a) [39.71 (48.25) nmol/L vs*.* 38.17 (33.34) nmol/L, *p* = 0.005] in CHD group were significantly higher than those in the control group, whereas the apoA1 and HDL-C levels were lower. The distribution of *LPA* the two SNP genotypes were both significantly different between the CHD group and control group (Table 1). Rs6415084 (CT/TT) and rs12194138 (AT/TT) genotypes were more prone to CHD than CC and AA genotypes (17.25% vs*.* 11.44%, *p*. < 0.001; 6.81% vs*.* 4.54%, *p* = 0.027).

**Table 1:** Baseline characteristics of all subjects.

Variables	Total	CHD group	Control group	*p* value
N (%)	2202	1101 (50.00%)	1101 (50.00%)	–
Age (years)	54.28 ± 11.01	54.98 ± 10.19	53.53 ± 11.32	0.187
Male (%)	1281 (58.17%)	660 (59.95%)	621 (56.40%)	0.101
BMI (kg/m^2^)	26.05 ± 3.10	26.14 ± 2.92	25.83 ± 3.22	0.181
Diabetes	569 (25.84%)	301 (27.34%)	268 (24.34%)	0.119
Hypertension	1531 (69.53%)	781 (70.94%)	750 (68.12%)	0.165
Smoking	612 (27.79%)	289 (26.25%)	323 (29.34%)	0.116
Consumers of alcohol	669 (30.38%)	345 (31.34%)	324 (29.43%)	0.354
**Laboratory variables**				
FPG (mmol/L)	6.15 ± 2.02	6.22 ± 2.07	6.06 ± 1.81	0.224
HbA1c (%)	5.93 ± 1.41	6.03 ± 1.49	5.75 ± 1.40	0.142
ApoB (mg/dL)	82.05 ± 28.54	88.55 ± 29.71	76.65 ± 28.12	< 0.001
ApoA1 (mg/dL)	148.78 ± 32.98	130.22 ± 30.13	175.85 ± 34.85	< 0.001
Total cholesterol (mmol/L)	4.43 (1.36)	4.51 (1.50)	4.39 (0.96)	0.155
Triglycerides (mmol/L)	1.33 (0.92)	1.35 (0.88)	1.32 (0.95)	0.469
HDL-C (mmol/L)	1.06 (0.47)	0.86 (0.31)	1.32 (0.40)	< 0.001
LDL-C (mmol/L)	2.68 (0.87)	2.79 (1.36)	2.65 (0.63)	< 0.001
sdLDL-C (mmol/L)	0.73 (0.35)	0.77 (0.48)	0.71 (0.31)	0.002
hs-CRP (mg/L)	2.43 (3.27)	4.01 (3.82)	2.10(1.56)	0.021
HCY (μmol/L)	10.68 (6.97)	12.16 (10.02)	9.58 (5.48)	< 0.001
Lp(a) (nmol/L)	39.33 (38.49)	39.71 (48.25)	38.17 (33.34)	0.005
**Minor allele frequency, N (%)**				
SNP rs6415084	316 (14.35%)	190 (17.25%)	126 (11.44%)	< 0.001
SNP rs12194138	125 (5.68%)	75 (6.81%)	50 (4.54%)	0.027

Data are reported as means ± SD or n(%), median (interquartile ranges). SD: Standard deviation.

Statistical analysis was performed with the student t test or Mann–Whitney U test and with Chi-square test for categorical variables.

*BMI* Body mass index, *HbA1c* Hemoglobin A1c, *apoB* Apolipoprotein B, *apoA1* Apolipoprotein A1, *HDL-C* High density lipoprotein cholesterol, *LDL-C* Low density lipoprotein cholesterol, *sdLDL-C* Small dense low-density lipoprotein cholesterol, *Hs-CRP* Hypersensitive C-reactive protein, *HCY* Homocysteine, *Lp(a)* Lipoprotein(a).

### The serum Lp (a) level and the prevalence of LPA SNPs were associated with the severity of CHD

The patients with CHD were then classified into single-vessel (n = 287), two-vessel (n = 310), and multiple-vessel disease (n = 504) groups (Table 2). We found a significant higher in the Lp(a) levels in multiple-vessel diseases group [31.40 (41.32) nmol/L vs. 40.09 (49.55) nmol/L vs. 45.23 (49.01) nmol/L, *p* < 0.001]. The distribution of *LPA* SNPs is shown (Table 2) and both of them were significantly different among multiple-vessel diseases groups. In patients with CHD, rs6415084 (CT/TT) genotype carriers are more prone to multiple-vessel obstruction (13.59% vs*.* 18.84% vs*.* 20.83%, *p* = 0.014). Rs12194138 (AT/TT) genotype carriers are more likely to have more than two-vessel of obstruction (3.14% vs*.* 10.00% vs*.* 6.94%, *p* = 0.004).

**Table 2:** Baseline characteristics of patients with multi vessel coronary artery disease.

Variables	1 vessel	2 vessels	≥ 3 vessels	*p* value
N (%)	287 (26.07%)	310 (28.16%)	504 (45.78%)	–
Age (years)	54.52 ± 11.01	55.74 ± 11.32	54.41 ± 10.27	0.592
Male (%)	169 (58.89%)	181 (58.39%)	310 (61.51%)	0.619
BMI (kg/m^2^)	25.92 ± 3.15	26.04 ± 3.38	26.28 ± 3.61	0.197
Diabetes	76 (26.48%)	89 (28.71%)	136 (26.98%)	0.806
Hypertension	189 (65.85%)	226 (72.90%)	366 (72.62%)	0.088
Smoking	88 (30.66%)	84 (27.10%)	117 (23.21%)	0.067
Consumers of alcohol	81 (28.22%)	96 (30.97%)	168 (33.33%)	0.325
**Laboratory variables**				
FPG (mmol/L)	6.52 ± 2.31	5.98 ± 2.06	5.91 ± 2.04	0.080
HbA1c (%)	6.14 ± 1.43	6.01 ± 1.35	5.97 ± 1.42	0.202
ApoB (mg/dL)	69.45 ± 27.23	85.53 ± 21.70	102.19 ± 28.79*^#^	< 0.001
ApoA1 (mg/dL)	135.42 ± 25.40	122.68 ± 29.34*	119.14 ± 32.53*	< 0.001
Total cholesterol (mmol/L)	3.66 (1.31)	4.24 (1.31)*	5.21 (1.82)*^#^	< 0.001
Triglycerides (mmol/L)	1.31 (0.76)	1.32 (0.58)	1.41 (0.94)	0.325
HDL-C (mmol/L)	0.88 (0.35)	0.84 (0.30)	0.86 (0.27)	0.378
LDL-C (mmol/L)	2.26 (1.12)	2.70 (0.99)*	3.38 (1.41)*^#^	< 0.001
sdLDL-C (mmol/L)	0.59 (0.23)	0.74 (0.26)*	1.04 ( 0.51)*^#^	< 0.001
hs-CRP (mg/L)	4.59 (5.13)	4.10 (6.33)	3.10 (5.97)*^#^	0.001
HCY (μmol/L)	11.85 (8.08)	12.75 (12.74)	12.17 (12.25)	0.271
Lp(a) (nmol/L)	31.40 (41.32)	40.09 (49.55)*	45.23 (49.01)*^#^	< 0.001
**Minor allele frequency, N (%)**				
SNP rs6415084	39 (13.59%)	46 (18.84%)	105 (20.83%)*^#^	0.014
SNP rs12194138	9 (3.14%)	31 (10.00%)*	35(6.94%)*	0.004

Data are reported as means ± SD or n (%), median (interquartile ranges). SD: Standard deviation.

Statistical analysis was performed with the ANOVA or Kruskal–Wall test and and with Chi-square test for categorical variables.

*BMI* Body mass index, *HbA1c* Hemoglobin A1c, *apoB* Apolipoprotein B, *apoA1* Apolipoprotein A1, *HDL-C* High density lipoprotein cholesterol, *LDL-C* Low density lipoprotein cholesterol, *sdLDL-C* Small dense low-density lipoprotein cholesterol, *Hs-CRP* Hypersensitive C-reactive protein, *HCY* Homocysteine, *Lp(a)* Lipoprotein(a).

*p < 0.05 compared with the 1 vessel group.

^#^*p* < 0.05 compared with the 2 vessels group.

The patients were also divided into three groups based on the GS tercile: low GS (≤ 25, n = 378), intermediate GS (26–41, n = 354), and high GS (≥ 42, n = 369) group (Table 3). The results showed that the level of serum Lp(a) in high GS group and Intermediate GS were significantly higher than Low GS groups [32.72 (41.33) nmol/L vs. 42.41 (56.80) nmol/L vs. 47.63 (58.48) nmol/L, *p* < 0.001]. Similarly, the number of *LPA* SNPs rs6415084 (CT/TT) and rs12194138 (AT/TT) genotype carriers in the high GS group was significantly higher than Low GS groups (14.29% vs*.* 16.38% vs*.* 21.14%, *p* = 0.040; 4.50% vs*.* 6.50% vs*.* 9.49%, *p* = 0.025).

**Table 3:** Baseline characteristics of Gensini score in patients with coronary heart disease.

Variables	Low GS	Intermediate GS	High GS	*p* value
N (%)	378 (34.33%)	354 (32.15%)	369 (33.51%)	–
Age (years)	54.84 ± 10.34	56.05 ± 10.14	54.71 ± 10.06	0.491
Male (%)	213 (56.35%)	222(62.71%)	225 (60.98%)	0.189
BMI (kg/m^2^)	26.11 ± 3.25	25.98 ± 3.13	26.42 ± 3.33	0.452
Diabetes	117 (30.95%)	87 (24.58%)	97 (26.29%)	0.132
Hypertension	257 (67.99%)	262 (74.01%)	262 (71.00%)	0.200
Smoking	106 (28.04%)	100 (28.25%)	83 (22.49%)	0.132
Consumers of alcohol	107 (28.31%)	124 (35.03%)	114 (30.89%)	0.135
**Laboratory variables**				
FPG (mmol/L)	6.32 ± 2.51	6.06 ± 2.37	5.98 ± 1.91	0.486
HbA1c (%)	6.18 ± 1.43	5.95 ± 1.42	6.04 ± 1.35	0.174
ApoB (mg/dL)	73.66 ± 27.06	88.54 ± 22.02*	105.31 ± 30.52*^#^	< 0.001
ApoA1 (mg/dL)	126.83 ± 29.37	123.47 ± 31.10	132.42 ± 27.07	0.057
Total cholesterol (mmol/L)	3.97 (1.19)	4.45 (1.34)*	5.22 (1.94)*^#^	< 0.001
Triglycerides (mmol/L)	1.15 (0.76)	1.23 (0.57)	1.59 (1.01)*^#^	< 0.001
HDL-C (mmol/L)	0.90 (0.33)	0.87 (0.29)	0.83 (0.25)	0.332
LDL-C (mmol/L)	2.46 (1.29)	2.77 (0.95)*	3.54 (1.41)*^#^	< 0.001
sdLDL-C (mmol/L)	0.58 (0.62)	0.80 (0.50)*	1.09 (1.05)*^#^	< 0.001
hs-CRP (mg/L)	2.65 (6.01)	4.06 (7.16)	3.90 (7.12)	0.643
HCY (μmol/L)	11.86 (11.14)	12.61 (11.02)	12.99 (12.78)	0.138
Lp(a) (nmol/L)	32.72 (41.33)	42.41 (56.80)*	47.63 (58.48)*	< 0.001
**Minor allele frequency, N (%)**				
SNP rs6415084	54 (14.29%)	58 (16.38%)	78 (21.14%)*	0.040
SNP rs12194138	17 (4.50%)	23 (6.50%)	35 (9.49%)*	0.025

Data are reported as means ± SD or n(%), median (interquartile ranges). SD: Standard deviation.

Statistical analysis was performed with the ANOVA or Kruskal–Wall test and and with Chi-square test for categorical variables.

*BMI* Body mass index, *HbA1c* Hemoglobin A1c, *apoB* Apolipoprotein B, *apoA1* Apolipoprotein A1, *HDL-C* High density lipoprotein cholesterol, *LDL-C* Low density lipoprotein cholesterol, *sdLDL-C* Small dense low-density lipoprotein cholesterol, *Hs-CRP* Hypersensitive C-reactive protein, *HCY* Homocysteine, *Lp(a)* Lipoprotein(a).

*p < 0.05 compared with the Low GS group.

^#^*p* < 0.05 compared with the Intermediate GS group.

### The effect of two SNPs on serum Lp(a) levels in Chinese Han people

The power values of SNPs rs6415084 and rs12194138 were 99% and 90%. The allele frequencies of SNPs rs6415084 and rs12194138 were 7.59% and 2.93%. The frequencies of the two tested variants did not deviate significantly in all subjects from the Hardy–Weinberg equilibrium: rs6415084, F = 2.630, *p* = 0.105; rs12194138, F = 2.502, *p* = 0.113.

Table 4 shows the relationship between *LPA* SNPs and serum Lp(a) levels. In *LPA* SNP rs6415084, the Lp(a) levels of rs6415084 (CC) genotype, rs6415084 (CT) genotype and rs6415084 (TT) genotype were different in all participants and CHD group (*p* < 0.001). In the control group, the Lp(a) levels of rs6415084 (CT/TT) genotype were higher than those of rs6415084 (CC) genotype (*p* < 0.001). In *LPA* SNP rs12194138, the Lp(a) levels of rs12194138 (AT/TT) genotype were higher than that of rs12194138 (AA) genotype in different groups (*p* < 0.001; *p* = 0.009; *p* < 0.001).

**Table 4:** Serum Lp(a) levels in all subjects carrying different *LPA* SNP genotypes.

rs6415084	All subjects			*p*
	CC (N = 1886)	CT (N = 298)	TT (N = 18)	
Lp(a) nmol/L	35.85 (31.33)	72.75 (78.20)*	157.91 (133.20)*^#^	< 0.001
	CHD group			
	CC (N = 911)	CT (N = 175)	TT (N = 15)	
Lp(a) nmol/L	37.74 (38.06)	81.83 (99.47)*	195.38 (61.87)*^#^	< 0.001
	Control group			
	CC (N = 975)	CT (N = 123)	TT (N = 3)	
Lp(a) nmol/L	34.16 (27.16)	70.16 (38.62)*	83.53 ( 36.28)*	< 0.001

rs12194138	All subjects			*p*
	AA (N = 2077)	AT (N = 121)	TT (N = 4)	
Lp(a) nmol/L	38.10 (38.44)	70.08 (32.47)*	168.12 (119.13)*^#^	< 0.001
	CHD group			
	AA (N = 1026)	AT (N = 71)	TT (N = 4)	
Lp(a) nmol/L	39.35 (50.53)	70.07 (33.57)*	168.12 (119.13)*^#^	0.009
	Control group			
	AA (N = 1051)	AT (N = 50)	TT (N = 0)	
Lp(a) nmol/L	36.84 (30.46)	70.92 (36.84)*	–	< 0.001

*p < 0.05 compared with the rs6415084 (CC) or rs12194138 (AA) group.

^#^*p* < 0.05 compared with the rs6415084 (CT) or rs12194138 (AT) group.

### Serum Lp (a) levels and LPA SNPs were associated with the risk of CHD.

In order to explore whether Lp(a) and *LPA* SNPs increase the risk of CHD, we conducted univariate and multivariate logistic regression analysis. All participants were divided into four groups according to the Lp(a) quartile level, and the presence and severity of CHD in individuals with different Lp(a) levels were assessed. In univariate logistic regression analysis, Lp(a) level was positively associated with the presence and severity of CHD (CHD group vs. control group: OR = 1.921, 95% CI: 1.102–3.121, *p* = 0.011; multiple-vessel disease group vs. single-vessel disease group: OR = 3.309, 95% CI: 2.293–5.030, *p* < 0.001; high GS group vs. low GS group: OR = 3.201, 95% CI: 1.234–8.09, *p* = 0.017) (Table 5). The multiple logistic regression analysis adjusted for age, sex, BMI, diabetes, hypertension, smoking, consumers of alcohol, FPG, HbA1c, ApoB, ApoA1, TC, TG, HDL-C, hs-CRP, and HCY, the level of Lp(a) remained to be independently associated with the presence and severity of CHD (CHD group vs. control group: OR = 1.793, 95% CI: 1.053–2.882, *p* = 0.043; multiple-vessel disease group vs. single-vessel disease group: OR = 1.941, 95% CI: 1.113–3.242, *p* = 0.027; high GS group vs. low GS group: OR = 2.641, 95% CI: 1.102–7.436, *p* = 0.040).

**Table 5:** Odd ratios of CHD, multiple-vessel disease and high GS in relation to quartiles of lipoprotein(a).

Variables	Lp(a), nmol/L			
	< 24.99	24.99–38.65	38.65–62.47	> 62.47
**CHD**				
Model 1^a^				
Odds ratio (95% CI)	1.00 (Ref.)	1.735 (0.931–2.877)	1.827 (1.063–2.832)	1.921 (1.102–3.121)
*p* value	–	0.106	0.024	0.011
Model 2^b^				
Odds ratio (95% CI)	1.00 (Ref.)	1.628 (0.621–3.214)	1.744 (0.798–2.012)	1.843 (1.058–2.732)
*p* value	–	0.658	0.195	0.041
Model 3^c^				
Odds ratio (95% CI)	1.00 (Ref.)	1.552 (0.658–2.661)	1.667 (0.723–2.985)	1.793 (1.053–2.882)
*p* value	–	0.505	0.449	0.043
**Multiple-vessel disease**				
Model 1^a^				
Odds ratio (95% CI)	1.00 (Ref.)	2.364 (1.345–4.155)	3.039 (1.506–6.132)	3.309 (2.293–5.030)
*p* value	–	0.003	0.002	< 0.001
Model 2^b^				
Odds ratio (95% CI)	1.00 (Ref.)	1.766 (0.660–4.725)	2.050 (0.720–5.836)	2.275 (1.318–3.927)
*p* value	–	0.257	0.179	0.003
Model 3^c^				
Odds ratio (95% CI)	1.00 (Ref.)	1.714 (0.754–4.821)	1.890 (0.705–5.091)	1.941 (1.113–3.242)
*p* value	–	0.267	0.206	0.027
**High GS**				
Model 1^a^				
Odds ratio (95% CI)	1.00 (Ref.)	2.321 (0.865–7.932)	2.943 (1.133–6.543)	3.201 (1.234–8.309)
*p* value	–	0.101	0.043	0.017
Model 2^b^				
Odds ratio (95% CI)	1.00 (Ref.)	2.358 (0.877–7.726)	2.727 (0.861–7.446)	2.766 (1.159–7.102)
*p* value	–	0.125	0.105	0.021
Model 3^c^				
Odds ratio (95% CI)	1.00 (Ref.)	1.784 (0.728–4.401)	2.025 (0.968–4.421)	2.641 (1.102–7.436)
*p* value	–	0.205	0.095	0.040

*Lp(a)* Lipoprotein(a), *GS* Gensini score, *CHD* Coronary heart disease, *CI* Confidence interval.

^a^Univariate model.

^b^Adjusted for age, sex, body mass index, diabetes, hypertension, smoking, consumers of alcohol.

^c^Additionally adjusted for hemoglobin A1c, fasting plasma glucose, apolipoprotein B, apolipoprotein A1, total cholesterol, triglycerides, high density lipoprotein cholesterol, hypersensitive C-reactive protein, and homocysteine.

Both of the *LPA* SNPs genotypes and allele frequency distributions are shown in Table 6. There were significant differences in genotype (rs6415084: CC vs. CT/TT; rs12194138: AA vs. AT/TT) and allele frequency (rs6415084: C vs. T; rs12194138: A vs. T) between CHD group and control group, multi vessel disease group and single vessel disease group, high GS group and low GS group (*p* < 0.05 for both). After adjusting for age, sex, BMI, diabetes, hypertension, smoking, consumers of alcohol, FPG, HbA1c, ApoB, ApoA1, TC, TG, HDL-C, hs-CRP and HCY were adjusted by multiple logistic regression analysis, it was found that both *LPA* SNPs rs6415084 (CT/TT ) and rs12194138 (AT/TT) were risk factors for CHD, and were positively associated with the severity of CHD (*LPA* SNPs rs6415084: CHD group vs. control group: OR = 1.577, 95% CI: 1.105–1.989, *p* = 0.004; multiple-vessel disease group vs. single-vessel disease group: OR = 1.613, 95% CI: 1.076–2.641, *p* = 0.030; high GS group vs. low GS group: OR = 1.580, 95% CI: 1.088–2.429, *p* = 0.024; *LPA* SNPs rs12194138: CHD group vs. control group: OR = 1.475, 95% CI: 1.040–3.002, *p* = 0.035; multiple-vessel disease group vs. single-vessel disease group: OR = 2.274, 95% CI: 1.060–5.148, *p* = 0.038; high GS group vs. low GS group: OR = 2.067, 95% CI: 1.101–4.647, *p* = 0.021). These data together indicated that the level of Lp(a) and the prevalence of *LPA* SNPs rs6415084 (CT/TT) and rs12194138 (AT/TT) is positively correlate with the severity of CHD.

**Table 6:** Relationship between two SNPs and coronary heart disease and its severity.

SNP	Groups	Genotype, n (%)			M ↔ m	Mm + mm ↔ MM	
		MM	Mm	mm	Crude OR (95% Cl)	Crude OR (95% Cl)	Adjusted OR (95% Cl)
**CHD**							
rs6415084	Control group	975 (88.6)	123 (11.2)	3 (0.2)	1.650 (1.312–2.073)	1.614 (1.266–2.057)	1.577 (1.105–1.989)
	CHD group	911 (82.7)	175 (15.9)	15 (1.4)	*p* < 0.001	*p* < 0.001	*p* = 0.004
rs12194138	Control group	1051 (95.4)	50 (4.6)	0 (0.0)	1.602 (1.118–2.294)	1.537 (1.063–2.220)	1.475 (1.040–3.002)
	CHD group	1026 (93.2)	71 (6.4)	4 (0.4)	*p* = 0.012	*p* = 0.027	*p* = 0.035
**Multiple-vessel disease**							
rs6415084	1 vessel	248 (86.4)	39 (13.6)	0 (0.0)	1.698 (1.161–2.483)	1.673 (1.107–2.466)	1.613 (1.076–2.641)
	≥ 3 vessels	399 (79.2)	99 (19.6)	6 (1.2)	*p* = 0.006	*p* = 0.013	*p* = 0.030
rs12194138	1 vessel	278 (96.9)	9 (3.1)	0 (0.0)	2.392 (1.146–4.993)	2.305 (1.092–4.867)	2.274 (1.060–5.148)
	≥ 3 vessels	469 (93.1)	33 (6.5)	2 (0.4)	*p* = 0.019	*p* = 0.024	*p* = 0.038
**High GS**							
rs6415084	Low GS	324 (85.7)	54 (14.3)	0 (0.0)	1.670 (1.167–2.389)	1.608 (1.098–2.355)	1.580 (1.088–2.429)
	High GS	291 (78.9)	72 (19.5)	6 (1.6)	*p* = 0.005	*p* = 0.016	*p* = 0.024
rs12194138	Low GS	361 (95.5)	17 (4.5)	0 (0.0)	2.360 (1.320–4.220)	2.225 (1.223–4.047)	2.067 (1.101–4.647)
	High GS	334 (90.5)	32 (8.7)	3 (0.8)	*p* = 0.004	*p* = 0.009	*p* = 0.021

Crude OR was determined by χ^2^ test, cases versus control subjects.

Adjusted OR was obtained on multivariate logistic regression after controlling for age, sex, body mass index, diabetes, hypertension, smoking, consumers of alcohol, hemoglobin A1c, fasting plasma glucose, apolipoprotein B, apolipoprotein A1, total cholesterol, triglycerides, high density lipoprotein cholesterol, hypersensitive C-reactive protein, and homocysteine.

M = C and m = T for single nucleotide polymorphism (SNP) rs6415084; M = A and m = T for single nucleotide polymorphism (SNP) rs12194138.

*CHD* Coronary heart disease, *CI* Confidence interval.

### Lp(a) levels, the prevalence of LPA SNPs and cardiovascular outcomes

**Figure 2:**
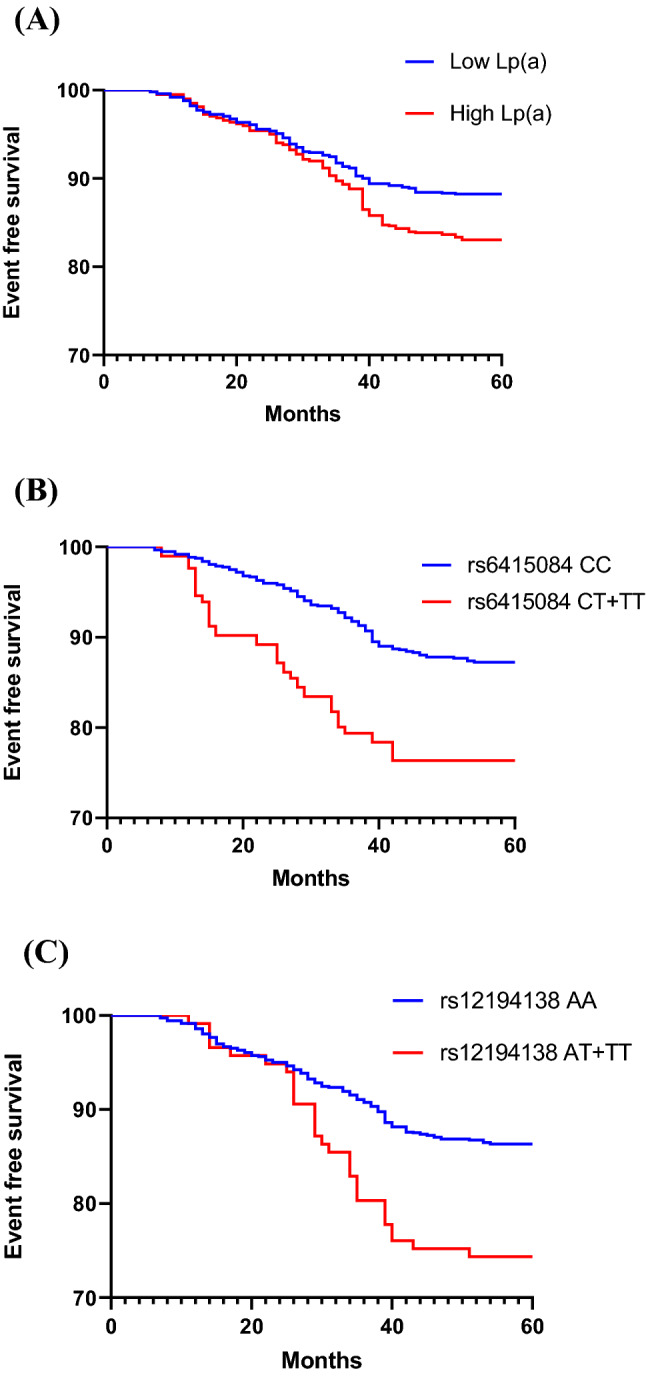
(**A**) Kaplan–Meier curves according to median value of Lp(a) (*p* = 0.001) in all subjects. (**B**, **C**) The cardiovascular event curves of SNPs rs6415084 (CT + TT) and rs12194138 (AT + TT) carriers were compared with SNPs rs6415084 (CC) and rs12194138 (AA) carriers (*p* < 0.001; *p* = 0.001) in all subjects.

**Figure 3:**
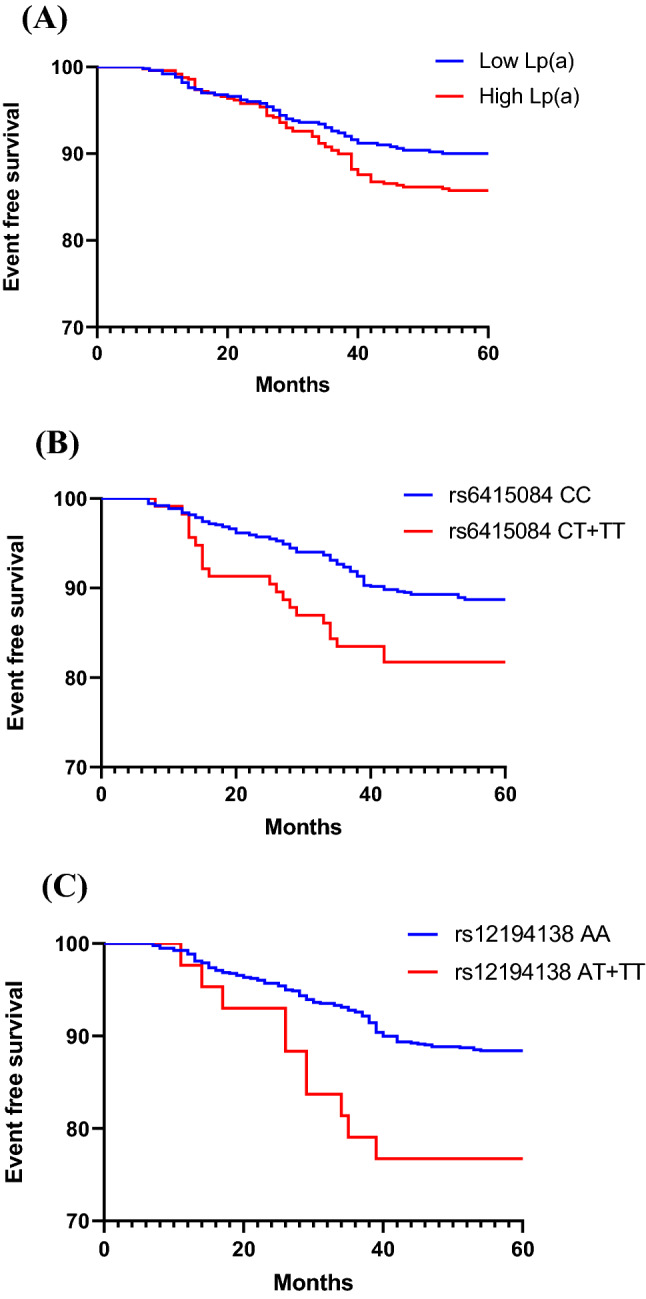
(**A**) Kaplan–Meier curves according to median value of Lp(a) (*p* = 0.044) in control group. (**B**, **C**) The cardiovascular event curves of SNPs rs6415084 (CT + TT) and rs12194138 (AT + TT) carriers were compared with SNPs rs6415084 (CC) and rs12194138 (AA) carriers (*p* = 0.022; *p* = 0.016) in control group.

**Figure 4:**
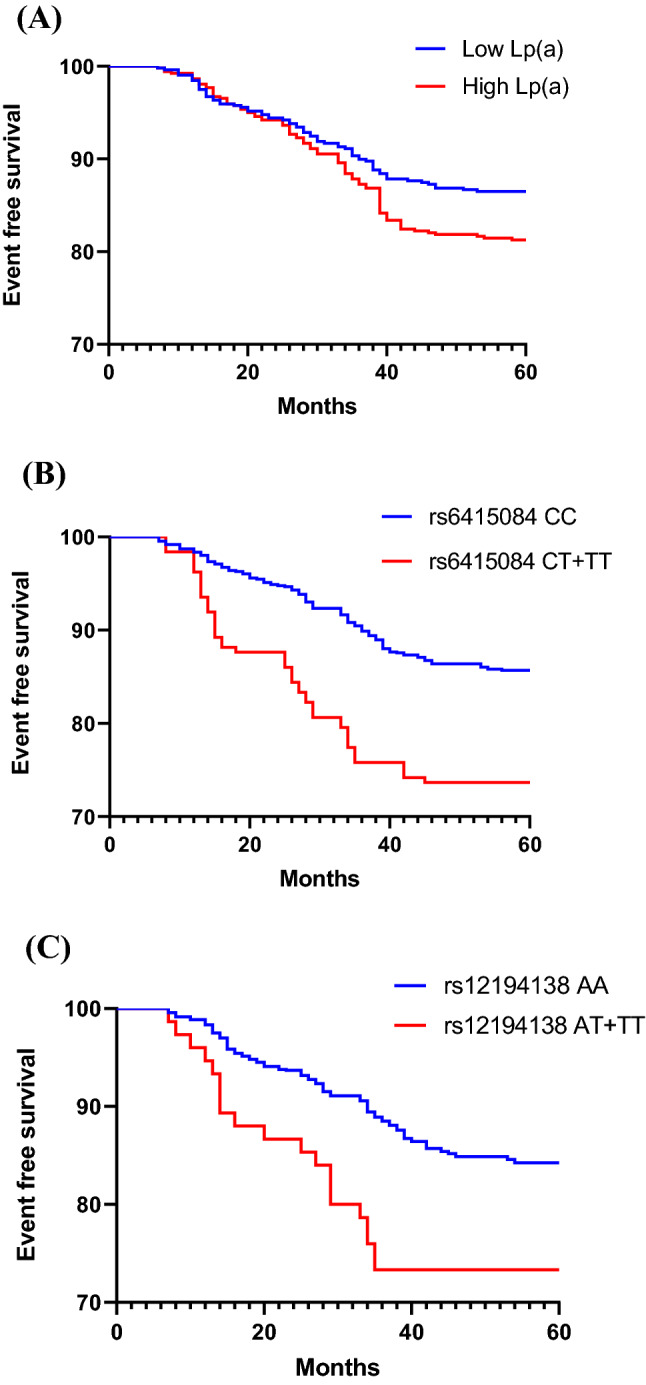
(**A**) Kaplan–Meier curves according to median value of Lp(a) (*p* = 0.011) in CHD patients. (**B**, **C**) The cardiovascular event curves of SNPs rs6415084 (CT + TT) and rs12194138 (AT + TT) carriers were compared with SNPs rs6415084 (CC) and rs12194138 (AA) carriers in CHD patients (*p* < 0.001; *p* = 0.007).

A total of 2042 individuals participated in the follow-up. During the follow-up period, 293 (14.35%) individuals had MACEs. All 2042 individuals were divided into high Lp(a) group and low Lp(a) group according to the median Lp(a), and Kaplan–Meier analysis was performed. The results showed that the incidence of MACEs in high Lp(a) group was higher than that in low Lp(a) group in all subjects (Fig. 2A) (HR = 1.459, 95% CI: 1.160–1.835, *p* = 0.001). In control group and CHD group, the results were the same (Figs. 3A, 4A) (HR = 1.443, 95% CI: 1.009–2.062, *p* = 0.044; HR = 1.473, 95% CI: 1.091–1.987, *p* = 0.011). The CHD and the control group were divided into two groups according to the median Lp(a), and the relationship between Lp(a) level and MACEs risk was studied. As shown in Tables 7 and 8, the total incidence of MACEs in the high Lp(a) group was significantly higher than that in the low Lp(a) group, both in the control group and the CHD group. These data indicate that Lp(a) level is associated with MACEs of all subjects.

**Table 7:** The relationship between Lp (a) level and the occurrence of MACEs in control group.

MACEs	Low Lp(a) (n = 502)	High Lp(a) (n = 499)	*p*
CHD	24 (4.78%)	33 (6.61%)	0.222
Cardiovascular deaths	0 (0.00%)	3 (0.60%)	0.124
Non-fatal MI	7 (1.39%)	11 (2.20%)	0.354
Non-fatal strokes	6 (1.20%)	5 (1.00%)	1.000
Heart failure	3 (0.60%)	3 (0.60%)	1.000
Hospitalized unstable angina	10 (1.99%)	16 (3.21%)	0.240
Total	50 (9.96%)	71 (14.23%)	0.042

Statistical analysis was performed with Chi-square test for categorical variables.

*Lp(a)* Lipoprotein(a), *CHD* Coronary heart disease, *MACEs* Major cardiovascular events.

**Table 8:** The relationship between Lp(a) level and the occurrence of MACEs in CHD patients.

MACEs	Low Lp(a) (n = 518)	High Lp(a) (n = 523)	*p*
Cardiovascular deaths	9 (1.74%)	12 (2.29%)	0.660
Non-fatal MI	30 (5.79%)	45 (8.60%)	0.093
Non-fatal strokes	13 (2.51%)	17 (3.25%)	0.579
Heart failure	8 (1.54%)	13 (2.49%)	0.379
Hospitalized unstable angina	10 (1.93%)	15 (2.87%)	0.419
Total	70 (13.51%)	102 (19.50%)	0.010

Statistical analysis was performed with Chi-square test for categorical variables.

*Lp(a)* Lipoprotein(a), *CHD* Coronary heart disease, *MACEs* Major cardiovascular events.

Further study of *LPA* SNPs and cardiovascular outcomes showed that MACEs were significantly higher in individuals with *LPA* SNPs rs6415084 (CT/TT) and rs12194138 (AT/TT) genotype carriers than CC and AA genotype carriers in all subjects (Fig. 2B,C) (HR = 2.499, 95% CI: 1.783–3.502, *p* < 0.001; HR = 2.565, 95% CI: 1.545–4.257, *p* = 0.001). In the control group, the results were consistent with the test results of all subjects (Fig. 3B,C) (HR = 1.949, 95% CI: 1.099–3.456, *p* = 0.022; HR = 3.087, 95% CI: 1.238–7.698, *p* = 0.016). The results were the same in the CHD group (Fig. 4B,C) (HR = 2.441, 95% CI: 1.628–3.661, *p* < 0.001; HR = 2.298, 95% CI: 1.254–4.211, *p* = 0.007). As shown in Tables 9 and 10, the total incidence of MACEs in *LPA* SNPs rs6415084 (CT/TT) and rs12194138 (AT/TT) genotype carriers was significantly higher than that in CC and AA genotype carriers, and there were differences in the non-fatal MI and hospitalized unstable angina of SNPs rs6415084 (CT/TT vs. CC) in the CHD group in addition to the total incidence. These results indicate that the *LPA* SNPs rs6415084 (CT/TT) and rs12194138 (AT/TT) genotype are related to the occurrence of cardiovascular events in the future.

**Table 9:** SNPs rs6415084 and rs12194138 were associated with MACEs in control group.

MACEs	SNP rs6415084		*p*	SNP rs12194138		*p*
	MM (886)	Mm + mm (115)		MM (958)	Mm + mm (43)	
CHD	47 (5.30%)	10 (8.70%)	0.137	52 (5.43%)	5 (11.63%)	0.092
Cardiovascular deaths	2 (0.23%)	1 (0.87%)	0.307	3 (0.31%)	0 (0.00%)	1.000
Non-fatal MI	17 (1.92%)	1 (0.87%)	0.711	16 (1.67%)	2 (4.65%)	0.179
Non-fatal strokes	10 (1.13%)	1 (0.87%)	1.000	11 (1.15%)	0 (0.00%)	1.000
Heart failure	4 (0.45%)	2 (1.74%)	0.144	5 (0.52%)	1 (2.33%)	0.232
Hospitalized unstable angina	20 (2.26%)	6 (5.22%)	0.108	24 (2.51%)	2 (4.65%)	0.308
Total	100 (11.29%)	21 (18.26%)	0.046	111 (11.59%)	10 (23.26%)	0.030

M = C and m = T for single nucleotide polymorphism (SNP) rs6415084; M = A and m = T for single nucleotide polymorphism (SNP) rs12194138.

**Table 10:** SNPs rs6415084 and rs12194138 were associated with MACEs in CHD patients.

MACEs	SNP rs6415084		*p*	SNP rs12194138		*p*
	MM (860)	Mm + mm (181)		MM (967)	Mm + mm (74)	
Cardiovascular deaths	15 (1.74%)	6 (3.31%)	0.237	19 (1.96%)	1 (1.35%)	1.000
Non-fatal MI	56 (6.51%)	21 (11.60%)	0.027	67 (6.93%)	8 (10.81%)	0.238
Non-fatal strokes	21 (2.44%)	8 (4.42%)	0.141	26 (2.69%)	4 (5.41%)	0.159
Heart failure	13 (1.51%)	5 (2.76%)	0.221	16 (1.65%)	3 (4.05%)	0.147
Hospitalized unstable angina	18 (2.09%)	9 (4.97%)	0.037	24 (2.48%)	4 (5.41%)	0.132
Total	123 (14.30%)	49 (27.07%)	< 0.001	152 (15.72%)	20 (27.03%)	0.022

M = C and m = T for single nucleotide polymorphism (SNP) rs6415084; M = A and m = T for single nucleotide polymorphism (SNP) rs12194138.

## Discussion

Recently, the role of Lp(a) in cardiovascular diseases has attracted more and more attention^15,19,20^. The association between Lp(a) and CHD, which is independent of traditional cardiovascular risk factors, has been known for many years^4,21^. It is based on findings mainly from studies of healthy participants in the general population and investigations of patients with CHD^22^. This study demonstrates that Lp(a) level and the prevalence of *LPA* SNPs are associated with the risk and severity of CHD, and *LPA* SNPs rs6415084 and rs12194138 are significantly associated with serum Lp(a) levels. That the increase of Lp(a) level was the key variable to predict CHD risk, and the role of *LPA* SNPs rs6415084, rs12194138 and Lp(a) level in predicting CHD risk was clarified. Most of the previous data on *LPA* and Lp(a) were studied in Caucasian populations in Europe^23–25^. However, there are few studies on the relationship between *LPA* and Lp(a) and the risk of coronary heart disease in Chinese Han population. At the same time, our study also predicted the occurrence of MACEs between rs6415084 and rs12194138 in Chinese Han population, so as to better study the role of Lp(a) level and *LPA* SNPs in predicting future MACEs of different nationalities.

We demonstrated that the rs6415084 and rs12194138 polymorphisms were significantly more frequent in patients with CHD than in healthy subjects. A higher risk of CHD was observed for the rs6415084 CT/TT and the rs12194138 AT/TT heterozygotes and homozygotes polymorphism carriers. The study of Lee et al.^15^, showed that the *LPA* SNPs was associated with the size of apolipoprotein (a) isoforms and the serum level of Lp(a) in different ethnicity. Clarke et al.^3^ showed that *LPA* SNPs rs10455872 and rs3798220 were strongly associated with increased level of Lp(a), a reduced copy number in *LPA*, and a small Lp(a) size. Lanktree et al.^14^ reported that SNPs rs6415084, located in the 5′ haplotype block and associated with KIV-2 copy number. Tolbus et al.^18^ reported that *LPA* SNPs rs12194138 were not associated with KIV-2 number of repeats. The other study including that identified SNPs in the *LPA* gene that had an association with the risk of CAD in diabetic patients^26^ (OR = 1.25, 95CI: 1.19–1.31, *p* = 3.92E-21). The results of Sang-Rok Lee et al. support our findings on the *LPA* SNPs and CHD.

Consistent with the role of Lp(a) in predicting future MACEs, it can be concluded that higher Lp(a) levels are primarily important to CHD risk prediction^27,28^. The increase of plasma Lp(a) level can promote thrombosis, and there is a correlation between Lp(a) and atherosclerotic stenosis. The same results are presented in some recent research reports^29^.

At present, most of the studies on *LPA* SNPs focus on the risk of CHD and atherosclerosis^30^. The prediction of future MACEs by SNPs is only seen in a few articles, and most of them focus on the European population^31^. Therefore, our study of the prediction of MACEs by two SNPs rs6415084 and rs12194138 in Chinese Han population, in order to better study the role of Lp(a) level and *LPA* SNPs in predicting future MACEs in different ethnicity. Our results are similar to those predicted by Gudbjartsson et al.^1^, for patients with diabetes mellitus with CHD in Iceland. CHD is a multifactorial disease, the combination of genetic variation and environmental factors may lead to phenotypic variation^32^. In our study, elevated Lp(a) level is an independent MACEs predictor; and some specific *LPA* SNPs variations may cause the increase of serum Lp(a), which also explains that SNPs rs6415084 and rs12194138 are independent MACEs predictors. Some studies have shown that *LPA* SNPs variation can increase Lp(a) level, but there is no direct correlation between *LPA* SNPs and MACEs^3,33^. The reason for this difference was not fully understood, but the discrepancy between the results of the studies may be caused by a variety of confounding factors, such as different population characteristics, study design, disease status, or confounding variables. Therefore, we used a large number of Han Chinese in this study. We not only found that serum Lp(a) levels and *LPA* SNPs variants were associated with the risk and severity of coronary heart disease, but they were independent predictors of MACEs.

Serum Lp(a) level is largely determined by the variation of *LPA* in many populations. Genetic variation of *LPA* is directly related to the risk of cardiovascular disease. The reason and mechanism of *LPA* variants rs6415084 and rs12194138 and Lp(a) level increase remains unclear^2,14^. The mechanism of increased Lp (a) lipoprotein level increasing the risk of coronary heart disease is unclear, which may involve LDL lipoprotein cholesterol^34^, inhibition of plasminogen to plasmin^35^, inhibition of tissue factor expression^36^, or carrying pro-inflammatory oxidized phospholipids^37^. From our results, we found that the level of Lp(a) is positively associated with the risk and severity of CHD.

Besides, the present study has several limitations. First, only two centers were involved in the research, which might have led to selective biases in the data results, and some of the conclusions should be verified in larger multicenter studies. Second, we have only studied two SNPs and the power value of SNPs rs12194138 is low. In our future work, we will increase more SNPs and sample size. In addition, CAG results lack of centralized core laboratory for angiography analysis. However, our data still provide the incidence rate and severity of CHD increase with the increase of serum Lp(a) level. In addition, the increased serum Lp(a) level and SNPs rs6415084 and rs12194138 variants will also increase the risk of cardiovascular events in the future.

## Conclusion

In conclusion, our data support the association of elevated serum Lp(a) levels and *LPA* SNPs rs6415084, rs3798220 variants with the risk and severity of coronary heart disease, and the prediction of future cardiovascular events.

## Data Availability

The datasets used and/or analyzed during the current study are not publicly available but are available from the corresponding author on reasonable request.
